# Regulation of Neuronal Senescence by *Srebf2* and *Zmiz1* Reveals Mechanisms of Aging-Related Neurodegeneration

**DOI:** 10.3390/biology15120938

**Published:** 2026-06-16

**Authors:** Zhiyu Deng, Jiale Chen, Jing Li, Xiaoman Luo, Qingming Luo, Miao Ren, Xiangning Li

**Affiliations:** 1 Key Laboratory of Biomedical Engineering of Hainan Province, School of Biomedical Engineering, Hainan University, Haikou 570228, China; 2 Department of Medical 3D Printing Center, The First Affiliated Hospital of Soochow University, Suzhou 215006, China; 3 HUST-Suzhou Institute for Brainsmatics, Jiangsu Industrial Technology Research Institute, Suzhou 215123, China

**Keywords:** neuronal senescence, neurodegeneration, cholesterol metabolism, cholinergic synapse, transcriptomic profiling, basal forebrain neurons

## Abstract

Neuronal senescence-like states are increasingly implicated in age-related neurodegeneration, yet the neuron-intrinsic regulators that drive these phenotypes remain poorly defined. Guided by prior transcriptomic analysis of aged basal forebrain cholinergic neurons, we investigated *Srebf2* and *Zmiz1* using primary basal forebrain neuronal cultures with adeno-associated virus-mediated gain- and loss-of-function, quantitative immunocytochemistry, and low-input transcriptomic profiling of fluorescence-activated cell sorting-isolated neurons. Both perturbation strategies produced the expected directional changes in target transcripts. Overexpression of either gene increased the other, whereas knockdown did not elicit reciprocal suppression, indicating asymmetric regulatory coupling. Phenotypically, *Srebf2* showed a bidirectional association with senescence-like changes, as both overexpression and knockdown increased p16 and p21, whereas *Zmiz1* acted more directionally, with overexpression increasing and knockdown reducing these markers. Transcriptomic profiling revealed broad direction-dependent remodeling, including a set of 55 genes that changed concordantly across perturbation directions. Pathway analysis further showed specialization, with *Zmiz1* preferentially associated with an Alzheimer’s disease-related signature and *Srebf2* more strongly linked to cholinergic synapse programs. Together, these findings identify *Srebf2* and *Zmiz1* as coupled but non-equivalent regulators of a neuronal senescence-like program.

## Simple Summary

As people age, some brain cells begin to show stress-related changes that resemble cellular aging, and these changes may contribute to diseases such as Alzheimer’s disease. However, the genes inside neurons that drive this process are still not well understood. In this study, we focused on two candidate genes, *Srebf2* and *Zmiz1*, that were previously found to be increased in vulnerable aging brain neurons. We used cultured mouse basal forebrain neurons to ask whether changing these genes could influence aging-like cellular stress. We found that *Srebf2* was sensitive to changes in either direction, while higher *Zmiz1* tended to worsen aging-related changes and lower *Zmiz1* tended to reduce them. We also found that the two genes may influence one another, suggesting that they could form a reinforcing program under certain conditions. Further analyses suggested that these genes affect both shared and distinct cell functions. These findings improve our understanding of how gene programs inside neurons may contribute to brain aging and neurodegeneration, and they provide a useful experimental framework for identifying future targets to protect vulnerable neurons.

## 1. Introduction

Neuronal senescence, commonly defined as a persistent dysfunctional cellular state accompanied by DNA damage accumulation, proteostasis disruption, metabolic stress, and a pro-inflammatory secretory profile, has increasingly been recognized as a contributor to age-associated neurodegenerative disorders such as Alzheimer’s disease (AD) and Parkinson’s disease (PD) [1,2]. Recent work further supports a functional link between senescence-associated neuronal states and neurodegenerative vulnerability [3,4]. Despite these advances, the neuron-intrinsic regulators that connect aging-associated gene programs to senescence-like phenotypes, particularly within selectively vulnerable circuits, remain insufficiently defined. Among aging-sensitive circuits, the basal forebrain cholinergic system is especially relevant because progressive loss and dysfunction of cholinergic neurons are tightly linked to cognitive decline [5]. Connectome-level analyses further implicate dysfunction of the cholinotrophic basal forebrain system in dementia-related phenotypes, reinforcing the vulnerability of this circuit in aging-associated cognitive impairment [6]. At cellular resolution, single-cell transcriptomic studies have begun to map dysregulated pathways in aging neurons, and building on these observations, our prior single-cell analysis of aged basal forebrain cholinergic neurons identified *Srebf2* and *Zmiz1* as upregulated candidates potentially involved in aging-related neuronal decline [7]. However, how these factors causally engage senescence-associated phenotypes in neurons and whether they interact on shared or distinct pathways remain poorly understood.

Cholesterol metabolism represents a long-standing mechanistic axis in neuronal maintenance and degeneration, with implications that extend beyond lipid abundance to synaptic organization and endolysosomal function. Sterol regulatory element-binding transcription factor 2, encoded by *Srebf2*, is a core transcriptional regulator of cholesterol biosynthesis and lipid homeostasis and has been linked to neuronal health [8,9,10]. Dysregulated cholesterol biosynthesis has been proposed as a convergent driver of diverse neurodegenerative pathways, including altered proteopathic stress and synaptic injury in disease models [11,12,13], and emerging evidence suggests that *Srebf2*-centered cholesterol programs may interface with aging and neurodegenerative disease processes more broadly [14]. Together, these observations raise the possibility that the aging-associated factor *Srebf2* may be associated with senescence-like programs, potentially in a direction-dependent manner.

In parallel, zinc finger MIZ domain-containing protein 1, encoded by *Zmiz1*, functions as a transcriptional coactivator for multiple transcription factors, including p53, the androgen receptor, and NOTCH1, and has been primarily studied in neurodevelopmental disease and tumorigenesis [15,16]. *Zmiz1* variants have been implicated in syndromic neurodevelopmental disorders [15], while *Zmiz1*-related transcriptional or epigenetic dysregulation has also been reported in cancer and brain disease contexts [17,18]. Notably, as a transcriptional coactivator of NOTCH1, *Zmiz1* may intersect with AD-relevant mechanisms, raising the possibility that enhanced NOTCH1 transcriptional output could engage neuronal stress pathways [16]. These considerations suggest that *Zmiz1* may contribute to neuronal vulnerability through transcriptional reprogramming that is mechanistically distinct from, but potentially convergent with, the lipid-centered axis governed by *Srebf2*.

Despite increasing evidence connecting both cholesterol dysregulation and stress-responsive transcriptional programs to neurodegeneration, the relative contributions of *Srebf2* and *Zmiz1* to neuronal aging still remain unresolved within an experimental framework. Importantly, in vivo studies face inherent limitations when addressing neuron-intrinsic senescence mechanisms. Systemic metabolic states, glial interactions, and circuit-level compensation can obscure direct gene effects within neurons, while developmental compensation and constraints on spatiotemporal manipulation further complicate mechanistic dissection.

To overcome these barriers, we established an in vitro primary basal forebrain neuronal culture platform enabling controlled gain- and loss-of-function perturbations of *Srebf2* and *Zmiz1*. By integrating AAV-mediated overexpression or RNAi, quantitative immunocytochemistry of senescence and apoptosis-related markers, and low-input transcriptomic profiling with downstream differential expression, pathway enrichment, and network analyses, we aimed to define how *Srebf2* and *Zmiz1* modulate senescence-like phenotypes and to identify regulatory programs that may explain direction-dependent and gene-specific effects. This neuron-centered approach provides a framework to link candidate aging-upregulated regulators to functional readouts and disease-relevant transcriptional signatures, thereby informing potential strategies to mitigate aging-related neurodegenerative decline.

## 2. Methods

### 2.1. Experimental Animals

ChAT-Cre (stock No.: 018957) transgenic mice and LSL-H2B-GFP (stock No.: 036761) reporter mice were purchased from Jackson Laboratory. These two strains were crossed to generate double-positive ChAT-Cre × LSL-H2B-GFP hybrid mice for low-input transcriptomic profiling experiments in this study. In these hybrid mice, the nuclei of cholinergic neurons were specifically labeled with green fluorescence. C57BL/6 mice used for immunohistochemistry experiments were bred and housed by Guangzhou Ruige Biotechnology Co., Ltd. (Guangzhou, China). All mice were housed in SPF-grade, temperature-controlled animal facilities with ad libitum access to food and clean water. The animal room was maintained under constant temperature and humidity, with a twelve-hour light/dark cycle. All animal experiments described in this study were approved by the Animal Ethics Committee of Hainan University and conducted in accordance with relevant guidelines and regulations.

### 2.2. Primary Neurons Culturing and Gene Expression Modulation

The basal forebrains were dissected from P0 mice for primary neuron culturing. P0 neonatal mice were anesthetized by hypothermia according to the approved animal protocol, followed by rapid decapitation and immediate dissection of the basal forebrain to minimize pain, suffering, and distress. The tissue was first digested using 0.25% trypsin-EDTA (Gibco, Grand Island, NY, USA) for 10 min, followed by 0.5 μg/mL DNase I (Roche, Basel, Switzerland) for an additional 10 min at 37 °C. A single-cell suspension was obtained by gentle trituration and filtration through a 40 μm cell strainer (Corning, Corning, NY, USA). The suspension was then centrifuged at 900 rpm for 10 min, and the supernatant was discarded. The cell pellet was resuspended in complete Neurobasal medium (Neurobasal, Gibco, Grand Island, NY, USA; supplemented with 2% B27, Gibco, Grand Island, NY, USA; and 0.5 mM GlutaMAX, Gibco, Grand Island, NY, USA), and cells were seeded onto PDL-coated coverslips at a density of 1000 cells/mm^2^. The culture medium was completely replaced within the first 24 h and subsequently half-changed twice per week.

Given its non-pathogenic nature and low immunogenicity, adeno-associated virus (AAV) was selected as the viral vector in this study. Gene regulation was achieved through both overexpression and interference approaches. To construct overexpression and knockdown viral vectors, the target gene sequences were obtained from the NCBI database. Suitable plasmid backbones were selected, and the gene fragments were inserted between two designated restriction sites using standard molecular cloning techniques. Recombinant AAV vectors were produced by BrainVTA (Wuhan) Co., Ltd. (Wuhan, China). at a final titer of 5 × 10^12^ vg/mL. At eleven days post primary neuron culture, either the knockdown viral vector (AAV-U6-shRNA1(target gene)-CMV-EGFP-pA) or the overexpression vector (rAAV-CMV-(target gene)-HA-WPRE-bGH pA) was added to the culture medium at an MOI of 1.6 × 10^5^. These recombinant AAV vectors delivered the modified plasmids, enabling gene-specific knockdown or overexpression. Transduction efficiency was calculated as the percentage of EGFP- or HA-positive cells among NeuN-positive neurons in randomly selected confocal fields.

### 2.3. Flow Cytometric Sorting and Low-Input Bulk RNA Sequencing

To perform low-input bulk RNA sequencing, neurons were separated from mixed primary cultures using flow cytometric sorting. We first prepared a single-cell suspension of transduced primary neuronal cultures. The culture medium from primary mouse basal forebrain neurons was removed, and the dishes were washed with calcium- and magnesium-free HBSS (Gibco, Cat. No. 14175095). The cells were then digested with 1 mg/mL papain and 0.025% trypsin-EDTA for six min at 37 °C. After enzymatic digestion, the solution was removed, and 150 μL of HBSS was added and pipetted to collect dissociated cells. The resulting cell suspension was filtered through a 40 μm cell strainer to obtain a single-cell suspension, which then underwent cell flow cytometry to separate individual neurons. Hoechst 33342 nuclear staining with a blue fluorescence was used to differentiate cell debris from intact viable cells during flow cytometric sorting. In the overexpression groups, GFP fluorescence from the ChAT-Cre × LSL-H2B-GFP reporter was used to enrich cholinergic neurons, whereas in the knockdown groups, GFP fluorescence derived from the EGFP carried by the RNAi vector and was used to identify shRNA-transduced neurons. Cells that were double-labeled with green and blue fluorescence were collected based on the designated grouping scheme (Table 1), with 30 cells collected per sample and five replicate samples for each group. In addition to single-gene perturbations, we included combined overexpression (OE-S-Z) and combined knockdown (Ri-S-Z) conditions to evaluate potential interactions between *Srebf2* and *Zmiz1* at the transcriptome level.

To extract total RNA, a cell lysis buffer was prepared and used immediately to lyse the cells collected after flow cytometric sorting. A double-negative group without any fluorescent labeling was included to help distinguish background signals from the target population during flow cytometry. The gated population in the experimental groups, absent in the double-negative group and positive for both fluorescent markers, was identified as cholinergic neurons in overexpression groups and transduced neurons in knockdown groups. The resulting sorted population represented approximately 0.5% of all detected fragments.

After collecting cells for sequencing, cDNA libraries were constructed using the SuperScript™ IV Single Cell/Low Input cDNA PreAmp Kit (Invitrogen, Cat. No. 11752048) according to the manufacturer’s instructions. Table 2 lists the software tools used for data processing and analysis in this study.

In this study, each sample generated approximately 6 Gb of sequencing data. For each experimental condition, five biological replicates were included. The expression matrices were normalized, followed by quality control assessments including the number of reads, the number of detected genes, and the proportion of mitochondrial gene expression. No significant outliers were observed across samples, indicating that the sequencing data were of high quality.

### 2.4. Differential Gene Expression Analysis

Differential expression analysis was performed to determine whether gene expression levels significantly differed between experimental groups using statistical hypothesis testing. The gene expression matrix consisted of non-zero integers, with genes as rows and samples as columns. Due to the nature of sequencing data, a large number of zero values are typically present for individual samples. Given the high sequencing depth (large n) and low expected count (p), gene expression distributions are commonly modeled using Poisson or negative binomial distributions. However, subsequent studies have shown that the mean and variance of read counts are not equal, making the negative binomial distribution a more appropriate model for differential expression analysis. DESeq2 (version 1.44.2), which utilizes a negative binomial distribution, was used to identify differentially expressed genes (DEGs). This method is well-suited for experiments with biological replicates. In this study, samples were divided into eight experimental groups, each with five biological replicates, thus meeting the requirements for DESeq2 analysis.

DESeq2 employs a normalization method designed to address two main sources of variation, which are differences in sequencing depth (library size) between samples and compositional differences in gene expression across samples. For each gene, the logarithmic mean across all samples is calculated, excluding genes with infinite values (i.e., read counts of zero). Log transformation reduces the influence of outliers and smooths the data distribution. Genes with zero counts are excluded to retain stably expressed genes. A new matrix is generated by subtracting the log mean of each gene from the corresponding values in the log-transformed expression matrix. The median of each sample’s adjusted log values is calculated (as medians are less sensitive to outliers than means). The exponential of each sample’s median is then computed to yield the size factor for normalization. Raw read counts are divided by the size factor to obtain the normalized expression matrix. Finally, expression levels between each experimental group and the control group were compared to compute the log_2_ fold changes and corresponding *p*-values for statistical significance.

For transcriptomic analyses, the overexpression and knockdown datasets were generated from related but non-identical neuronal populations. Overexpression samples were obtained from transduced cholinergic-enriched basal forebrain neurons using the ChAT-Cre × LSL-H2B-GFP reporter system, whereas knockdown samples were obtained from transduced basal forebrain neurons without cholinergic enrichment. Accordingly, downstream analyses emphasized within-dataset comparisons, and cross-dataset overlaps were interpreted cautiously. Therefore, shared signatures across datasets should be interpreted as context-conserved responses rather than exact inverses within an identical neuronal subtype.

### 2.5. Gene Set Enrichment Analysis

Gene Set Enrichment Analysis (GSEA) was performed to determine whether a predefined set of genes shows statistically significant, concordant differences between two biological states or phenotypes. This method evaluates whether the members of a given gene set are non-randomly distributed toward the top or bottom of a ranked list of genes, thereby indicating potential association with the phenotype of interest. The core steps of GSEA are as follows: (1) Gene ranking: all genes were ranked based on their correlation with the phenotype, such as fold change or signal-to-noise ratio. Genes highly upregulated in the experimental group appear at the top of the list, while downregulated genes are positioned at the bottom. (2) Enrichment Score (ES) calculation: ES quantifies the degree to which a gene set is overrepresented at the extremes (top or bottom) of the ranked list. The ES is calculated by walking down the ranked list and increasing a running-sum statistic when a gene is in the gene set and decreasing it when it is not. The peak deviation from zero corresponds to the ES, indicating the degree of enrichment. A positive ES suggests enrichment at the top of the list, and a negative ES suggests enrichment at the bottom. (3) Significance assessment: the statistical significance of the observed ES was assessed using permutation testing. *p*-values were computed to determine whether the observed enrichment is likely to have occurred by chance. (4) Normalization of enrichment scores (NES): ES was normalized to account for differences in gene set sizes, yielding NES, which allows for comparison across gene sets. (5) Multiple hypothesis testing correction: to control for false positives due to multiple comparisons, the false discovery rate (FDR) was calculated. Only gene sets with FDR values below a specified threshold were considered significantly enriched.

### 2.6. Cellular Fluorescent Staining

Filipin III staining was used to detect cholesterol content by specifically interacting with free cholesterol. The cells were washed with PBS and incubated with a 0.1 mg/mL Filipin III working solution at room temperature for 30 min in the dark. Afterward, the cells were stained with 1 μg/mL propidium iodide (PI) for 5 min. Finally, the samples were analyzed using a confocal microscope.

Immunofluorescence staining was performed as follows: After washing with PBS, cells were fixed with 4% PFA at 4 °C for 2 h, followed by three PBS washes. Permeabilization and blocking were done using 0.03% triton X-100 and 5% BSA in PBS at 37 °C for 30 min. The samples were incubated overnight at 4 °C with primary antibodies, followed by three PBS washes, and with secondary antibodies at 37 °C for 2 h, again followed by three PBS washes. The types and concentrations of antibodies used were listed in Table 3. DAPI staining was applied at room temperature for 5 min at the end, followed by PBS washes. Finally, cells were mounted and imaged using a confocal microscope. The fluorescence intensity of transduced neuronal somata in confocal microscopic images was quantified and compared among experimental groups. Each experimental group included at least three coverslips subjected to the same treatment, and experiments were performed using three independent batches of primary neuronal cultures.

### 2.7. Statistical Analysis

For experiments involving comparisons among control, knockdown, and overexpression groups, statistical significance was assessed using one-way analysis of variance (ANOVA) followed by Dunnett’s multiple comparisons test, with the corresponding control group as the reference. This approach was used for fluorescence intensity analyses in which each perturbation group was compared with its matched control. Adjusted *p* values from Dunnett’s test were used to determine statistical significance. For comparisons involving only two groups, an unpaired two-tailed Student’s *t*-test was used. Data are presented as mean ± SD unless otherwise indicated. Error bars indicate SD. *p* < 0.05 was considered statistically significant.

## 3. Results

### 3.1. An AAV-Based Primary Basal Forebrain Neuron Model Enables Efficient Bidirectional Gene Perturbation and Phenotypic Readouts

**Figure 1:**
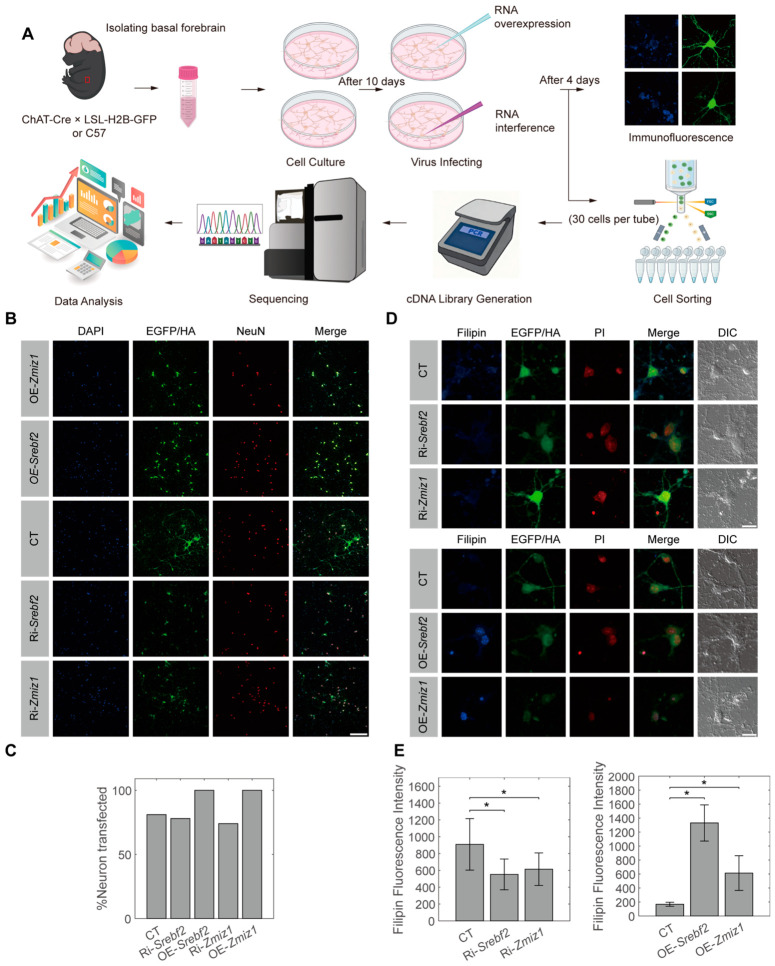
In vitro model using primary neurons for the study of gene modulation. (**A**) Workflow used in this study, including primary culturing, immunofluorescence, cell sorting, sequencing, and data analysis. (**B**) Virus vectors used in this study transduced primary neurons with high efficiency and without obvious morphological deterioration. Scale bar represents 200 μm. (**C**) Transduction efficiencies of all viruses used in this study were over 80% transduction efficiency, and some were close to 100%. (**D**) Fluorescence staining using Filipin showing the cholesterol content in primary neurons. Scale bar represents 50 μm. (**E**) The cellular cholesterol contents decreased when either gene was knocked down and increased when either gene was overexpressed. Statistical significance was assessed using one-way ANOVA followed by Dunnett’s multiple comparisons test against the corresponding control group. Significance (*p* < 0.05) is denoted using an asterisk.

To investigate the roles of *Srebf2* and *Zmiz1* in neuronal aging, we established a culture system that integrates mouse breeding, in vitro primary culturing, viral transduction, immunocytochemistry, and downstream analyses, including flow cytometry for transduced cell sorting and low-input RNA sequencing (Figure 1A). Our viral vectors allowed simultaneous monitoring of transduction efficiency and neuronal viability (via EGFP fluorescence or HA tag) and precise modulation of target gene expression (*Srebf2* and *Zmiz1*). This integrated workflow enabled simultaneous monitoring of molecular pathways (e.g., *Srebf2*-regulated cholesterol biosynthesis) and aging-associated phenotypes (e.g., p16/p21 expression), enabling direct linkage of gene perturbation to cellular phenotypes and downstream transcriptional programs.

To achieve precise modulation of *Srebf2* and *Zmiz1*, we constructed four AAV vectors for RNA interference (RNAi) or overexpression. For RNAi, two independent shRNA sequences targeting *Srebf2* and *Zmiz1* were cloned into a pAAV-U6-shRNA vector, with non-targeting scrambled sequences as controls. These constructs underwent in vitro screening using mouse B16 cells. qPCR confirmed >70% knockdown efficiency for both genes (*Srebf2*: 75.4 ± 7.3%, *Zmiz1*: 74.3 ± 5.5%, SEM, *p* < 0.001), with no cross-reactivity. For overexpression, codon-optimized *Srebf2* and *Zmiz1* cDNA were cloned into a pAAV-CAG-HA vector, achieving overexpression of respective genes in target cells.

We next validated the performance of this platform in primary neurons. At DIV11, neurons were transduced with control, *Srebf2*, or *Zmiz1* perturbation viruses, and robust EGFP or HA expression was detected by DIV14. Reporter signals were broadly distributed in neuronal somata and neurites, and transduced cultures retained dense neuronal networks with no obvious morphological deterioration (Figure 1B). Quantification further showed that all viral constructs efficiently transduced the cultures, with more than 80% of NeuN+ neurons positive for EGFP or HA (Figure 1C).

To assess the functional consequences of modulating *Srebf2* and *Zmiz1*, we first measured cellular cholesterol levels using Filipin III staining (Figure 1D). In line with its established role as a master regulator of cholesterol biosynthesis, *Srebf2* overexpression (OE-*Srebf2*) robustly increased cholesterol content, whereas its knockdown (Ri-*Srebf2*) significantly reduced it (Figure 1E). Notably, manipulation of *Zmiz1*, a transcriptional coactivator not canonically recognized as a cholesterol-biosynthetic regulator, also altered Filipin-labeled cholesterol levels. *Zmiz1* overexpression increased, whereas *Zmiz1* knockdown decreased, neuronal Filipin intensity, although the magnitude of these changes was smaller than that observed after direct *Srebf2* perturbation (Figure 1E). These findings suggest that *Zmiz1* is functionally associated with neuronal cholesterol homeostasis. Given that *Zmiz1* is not a core component of the cholesterol biosynthetic machinery, this effect is likely to occur through indirect transcriptional or network-mediated mechanisms, potentially involving *Srebf2*-dependent pathways or broader changes in neuronal stress and maintenance programs.

Together, these results establish a robust in vitro platform for bidirectional gene perturbation in primary basal forebrain neurons. The combination of high transduction efficiency, preserved neuronal integrity, and functionally responsive cholesterol readouts indicates that this system is well suited for interrogating downstream senescence-associated phenotypes and transcriptional programs.

### 3.2. Perturbation of Srebf2 or Zmiz1 Differentially Reshapes Senescence Markers and Caspase Activation

**Figure 2:**
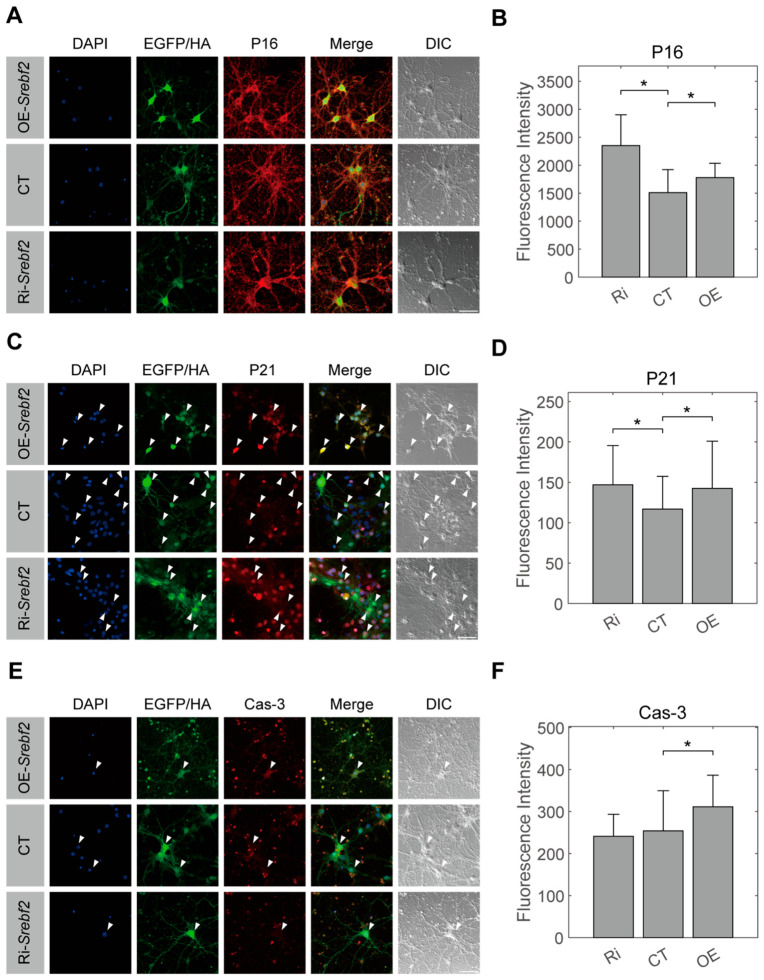
*Srebf2* modulates the expression of aging hallmarks. (**A**) P16 expression after *Srebf2* modulation. (**B**) P16 expression in transduced neurons increased in both *Srebf2* knockdown and overexpression groups. (**C**) P21 expression after *Srebf2* modulation. Arrowheads indicate transduced neuronal somata used for representative comparison and quantification; unmarked P21-positive cells outside the annotated neuronal population were not included in neuronal soma quantification. (**D**) P21 expression in transduced neurons increased in both *Srebf2* knockdown and overexpression groups. (**E**) Cleaved caspase-3 expression after *Srebf2* modulation. Arrowheads indicate neuronal somata. (**F**) Cleaved caspase-3 expression did not change significantly in *Srebf2* knockdown group and increased significantly in overexpression group. Statistical significance was assessed using one-way ANOVA followed by Dunnett’s multiple comparisons test against the corresponding control group. Significance (*p* < 0.05) is denoted using an asterisk. All scale bars represent 100 μm.

*Srebf2* perturbation produced a bidirectional senescence-marker response in primary basal forebrain neurons. Across all groups, P16 was predominantly retained in the neuronal cytoplasm and was not detected in non-neuronal cells (Figure 2A). Quantification showed that somatic P16 intensity increased in both Ri-*Srebf2* and OE-*Srebf2* neurons relative to control (Figure 2B). P21 displayed diffuse cytoplasmic staining in neurons as well as in other cell types (Figure 2C), and its somatic intensity was likewise increased in both Ri-*Srebf2* and OE-*Srebf2* neurons (Figure 2D). Thus, both downregulation and upregulation of *Srebf2* were associated with elevated senescence-marker levels. By contrast, cleaved caspase-3 showed a more selective response: although it was detected in neuronal somata and more strongly in other cell types (Figure 2E), its expression was significantly increased only in OE-*Srebf2* neurons, but not in Ri-*Srebf2* neurons (Figure 2F). Together, these data define a bidirectional senescence-associated response to *Srebf2* perturbation, with apoptotic signaling engaged primarily under overexpression conditions.

**Figure 3:**
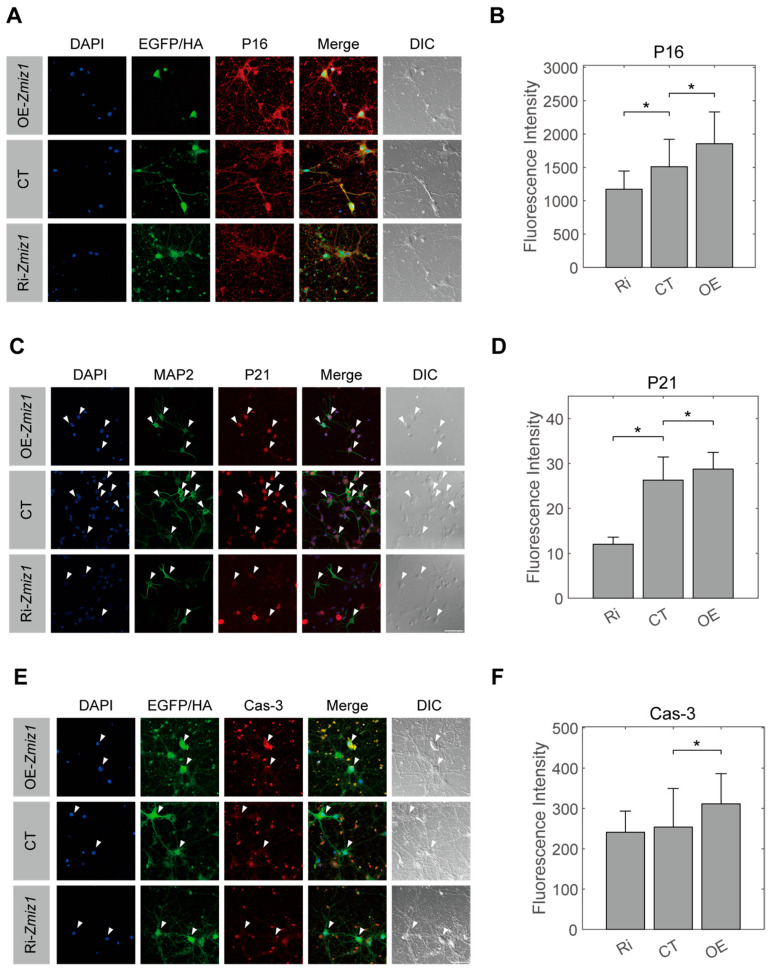
*Zmiz1* modulates the expression of aging hallmarks. (**A**) P16 expression after *Zmiz1* modulation. (**B**) P16 expression in transduced neurons decreased in *Zmiz1* knockdown group and increased in the overexpression group. (**C**) P21 expression after *Zmiz1* modulation. Arrowheads indicate neuronal somata. (**D**) P21 expression in transduced neurons decreased in *Zmiz1* knockdown group and increased in the overexpression group. (**E**) Cleaved caspase-3 expression after *Zmiz1* modulation. Arrowheads indicate neuronal somata. (**F**) Cleaved caspase-3 expression did not change significantly in *Zmiz1* knockdown group and increased significantly in overexpression group. Statistical significance was assessed using one-way ANOVA followed by Dunnett’s multiple comparisons test against the corresponding control group. Significance (*p* < 0.05) is denoted using an asterisk. All scale bars represent 100 μm.

In contrast to the bidirectional response observed for *Srebf2*, *Zmiz1* exhibited a directional modulation of senescence markers. Similar to the *Srebf2* dataset, P16 was present in neuronal somata and neurites but absent from other cell types (Figure 3A), whereas P21 showed diffuse cytoplasmic staining in neurons and relatively stronger labeling in non-neuronal cells (Figure 3C). However, unlike *Srebf2*, *Zmiz1* perturbation shifted both markers in opposite directions depending on the mode of manipulation: OE-*Zmiz1* increased somatic P16 and P21, whereas Ri-*Zmiz1* reduced both markers relative to control (Figure 3B,D). Cleaved caspase-3 followed the same overall trend, with increased expression in OE-*Zmiz1* neurons and no significant reduction in Ri-*Zmiz1* neurons (Figure 3E,F). These results indicate that *Zmiz1* abundance is directionally associated with senescence-marker expression, while its effect on caspase activation is more pronounced under overexpression.

Overall, these immunocytochemical readouts distinguish two senescence-associated marker response modes in basal forebrain neurons: a bidirectional p16/p21 response to *Srebf2* perturbation and a directional p16/p21 response to *Zmiz1* perturbation, while cleaved caspase-3 activation is preferentially observed under overexpression conditions.

### 3.3. Low-Input RNA-Seq Links Zmiz1 to Alzheimer’s Disease Programs and Srebf2 to Cholinergic Synapse Regulation

To define the transcriptional programs underlying the immunocytochemical phenotypes observed in primary basal forebrain neuronal cultures, we next performed low-input RNA sequencing on small pools of virally transduced neurons isolated by flow cytometry with 30 cells per sample. For the overexpression dataset, we profiled transduced basal forebrain cholinergic neurons collected using ChAT-Cre mice with H2B-GFP reporter, whereas for the knockdown dataset, we profiled transduced basal forebrain neurons without cholinergic enrichment. Because these datasets were generated from related but non-identical neuronal populations, differential expression, pathway enrichment, and network analyses were performed within each dataset relative to its corresponding control. Cross-dataset overlaps were interpreted conservatively as context-shared transcriptional responses rather than as exact inverse regulation within the same neuronal subtype. To capture both single-gene and combined perturbation effects, OE-S-Z and Ri-S-Z groups were included in the transcriptomic analysis.

**Figure 4:**
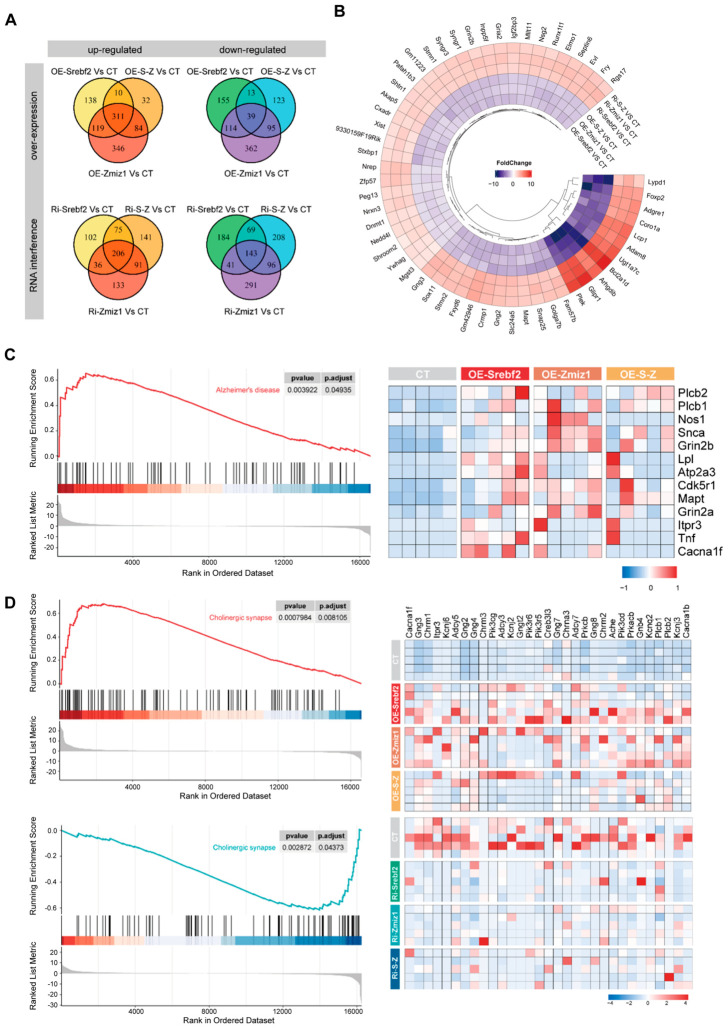
Differential gene expression analysis. (**A**) Venn diagram illustrating the overlap of differentially expressed genes among distinct experimental groups. (**B**) Circular heatmap showing log_2_ fold changes in the 55 concordantly regulated genes across overexpression and knockdown comparisons. The color scale represents log_2_ fold change, with red indicating upregulation and blue indicating downregulation. (**C**) GSEA of ranked genes after *Zmiz1* modulation showed enrichment of Alzheimer’s disease-related pathways. The left panel shows the GSEA result for OE-*Zmiz1* versus CT. The curve represents the enrichment score. Vertical lines indicate the ranking positions of pathway genes among all genes ranked in descending order by log_2_ fold change. The accompanying heatmap shows normalized expression levels of representative Alzheimer’s disease pathway-related genes across individual samples. Columns correspond to samples, rows correspond to genes, and the color scale represents normalized gene expression levels, with red indicating higher expression and blue indicating lower expression. Samples were grouped by experimental condition, and genes were displayed without clustering. (**D**) GSEA of ranked genes after *Srebf2* modulation showed enrichment of cholinergic synapse-related pathways. The left panels show GSEA results for OE-*Srebf2* versus CT and Ri-*Srebf2* versus CT. The curves represent enrichment scores. Vertical lines indicate the ranking positions of pathway genes among all genes ranked in descending order by log_2_ fold change. The accompanying heatmap shows normalized expression levels of representative cholinergic synapse-related genes across individual samples. Rows correspond to samples, columns correspond to genes, and the color scale represents normalized gene expression levels, with red indicating higher expression and blue indicating lower expression. Samples were grouped by experimental condition, and genes were displayed without clustering.

Low-input transcriptomic profiling revealed broad and direction-dependent remodeling of neuronal gene expression across perturbation groups. Among the overexpression conditions (OE-*Srebf2*, OE-*Zmiz1*, and OE-S-Z), 331 genes were commonly upregulated and 39 were commonly downregulated relative to control, whereas the knockdown conditions (Ri-*Srebf2*, Ri-*Zmiz1*, and Ri-S-Z) shared 206 upregulated and 143 downregulated genes (Figure 4A). Having established this global remodeling pattern, we next asked whether a conserved transcriptional response could be identified across perturbation directions. Indeed, 55 genes displayed consistent bidirectional behavior, increasing under overexpression and decreasing under knockdown across the perturbation groups (Figure 4B). This shared set included multiple transcripts implicated in neuronal vulnerability and neurodegenerative processes, such as *Mapt*, *Stmn1*, *Stmn2*, *Grin2b*, *Gria2*, and *Dnmt1*, supporting the biological relevance of a common downstream response program. The inclusion of combined perturbation groups allowed us to further evaluate whether *Srebf2* and *Zmiz1* act through simple additive effects or through partially convergent transcriptional programs. At the transcriptomic level, the combined overexpression and knockdown conditions contributed to the identification of gene sets commonly altered across single- and dual-gene perturbations (Figure 4A,B). Rather than indicating a uniform amplification of the single-gene effects, these comparisons suggest that dual perturbation primarily captures shared downstream responses and context-dependent convergence between *Srebf2*- and *Zmiz1*-associated programs.

We then examined whether this shared transcriptional remodeling was accompanied by gene-specific pathway specialization. For *Zmiz1*, the Alzheimer’s disease pathway was significantly enriched in OE-*Zmiz1* relative to control (Figure 4C), together with increased expression of representative pathway genes, including *Mapt*, *Snca*, *Grin2a*, *Grin2b*, and *Nos1*, in the corresponding heatmap. By contrast, *Srebf2* modulation was more strongly linked to cholinergic synapse-related programs, which were enriched in opposite directions, with upregulation in OE-*Srebf2* and downregulation in Ri-*Srebf2* (Figure 4D). Consistent with this directional pattern, the heatmap showed coordinated induction of multiple cholinergic synapse-related genes in OE-*Srebf2*, whereas Ri-*Srebf2* exhibited an overall downward trend, including significant suppression of selected signaling components such as Akt1, Prkaca, Camk2d, and Gng14. Together, these data indicate that although *Srebf2* and *Zmiz1* share a common transcriptional response axis, they diverge at the pathway level, with *Zmiz1* preferentially engaging an Alzheimer’s disease–associated signature and *Srebf2* more prominently affecting cholinergic synaptic programs.

### 3.4. Co-Expression Network Analysis Reveals Context-Dependent Coupling of Srebf2 and Zmiz1 and a Protective Module Inversely Associated with Senescence Markers

To move beyond pathway-level associations and examine how *Srebf2* and *Zmiz1* are embedded within coordinated gene programs, we next performed co-expression network analysis on the low-input transcriptomic datasets. Co-expression networks were constructed separately for the overexpression and knockdown datasets. This approach allowed us to identify modules linked to each perturbation and to determine whether the relationship between the two genes is reflected at the module level.

**Figure 5:**
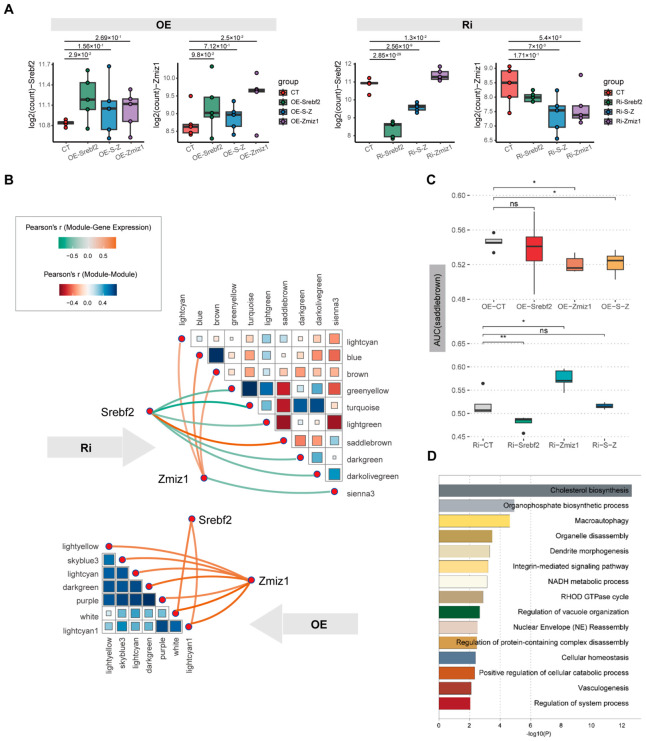
Gene interaction and network analysis. (**A**) The expression of target genes *Srebf2* and *Zmiz1* in all experimental groups. Individual data points correspond to biological replicates. The y-axis represents normalized expression levels. *p* values for intergroup comparisons are indicated above the lines. (**B**) Simplified correlation network of co-expression modules in the RNAi and overexpression datasets. Module–module correlations are shown as square matrices, and module–target gene correlations are shown as connecting lines between *Srebf2*/*Zmiz1* and their associated modules. Only significant correlations are displayed to improve readability. Color indicates the direction of Pearson’s correlation, and square or line intensity reflects correlation strength. Orange lines indicate positive module–target gene correlations, whereas green lines indicate negative correlations. (**C**) Differential AUC scores of the saddlebrown gene module across groups. Statistical significance was assessed using the Wilcoxon test. Significance levels are denoted as follows: ns, not significant (*p* > 0.05); *, *p* < 0.05; **, *p* < 0.01. (**D**) Top 15 enriched pathways of the saddlebrown gene module. The bar plot shows the top 15 enriched pathways. The x-axis represents the −log_10_ (*p* value).

To explore potential interactions between *Srebf2* and *Zmiz1*, we first confirmed the efficiency of viral perturbations by quantifying their mRNA levels. Both overexpression and knockdown produced the expected directional changes in the target transcripts (Figure 5A). Notably, overexpression of *Srebf2* was accompanied by increased *Zmiz1* expression, and overexpression of *Zmiz1* similarly elevated *Srebf2*, whereas knockdown of either gene did not reduce the other (Figure 5A). Together, these data reveal an asymmetric regulatory coupling pattern in which reciprocal induction occurs under overexpression but is not mirrored under knockdown conditions.

This analysis identified 39 modules in the overexpression condition and 31 modules in the knockdown condition (Figure 5B). In the overexpression network, *Srebf2* was associated with two modules (OE-white and OE-lightcyan1), while *Zmiz1* was linked to seven modules, including OE-white and OE-purple. In the knockdown network, *Srebf2* was primarily associated with a single module (Ri-saddlebrown), whereas *Zmiz1* correlated with three modules (Ri-lightcyan, Ri-blue, and Ri-brown) (Figure 5B). Consistent with a context-dependent regulatory architecture, *Srebf2* and *Zmiz1* converged on the same module under overexpression (OE-white) but mapped to distinct modules under knockdown (Ri-saddlebrown for *Srebf2* and Ri-blue for *Zmiz1*). These module-association patterns indicate that the coupling between *Srebf2* and *Zmiz1* is shared in the overexpression network but becomes more segregated in the knockdown network, revealing direction-dependent network organization. Functional enrichment analysis of additional *Srebf2*- and *Zmiz1*-associated modules further showed distinct biological themes across overexpression and RNAi networks, including chromatin/RNA-related processes, synaptic vesicle/SNARE-related functions, lipid-transport-related activity, and cytoskeletal or transcriptional regulatory processes (Supplementary Figure S1). Ri-saddlebrown was particularly notable among these modules because it was closely associated with *Srebf2* in the knockdown network and was enriched in neuronal maintenance-related processes. To further show the gene composition of these modules, the top 25 hub genes ranked by module membership (kME) are provided in Supplementary Figure S2.

Given that Ri-saddlebrown emerged as the major module associated with *Srebf2* specifically within the knockdown dataset, we next examined whether its activity varied systematically across experimental groups. Module-level activity, quantified by AUC scores, showed significant group-dependent differences (Figure 5C). Within the knockdown condition, Ri-*Srebf2* displayed reduced saddlebrown activity, whereas Ri-*Zmiz1* showed increased saddlebrown activity, and the combined knockdown group exhibited an intermediate pattern (Figure 5C). The enrichment score followed the same trend as the AUC score, supporting the robustness of the module activity changes. Interestingly, the AUC scores and enrichment scores inversely correlated with the expression levels of aging markers, further supporting an inverse association between the saddlebrown module, with which *Srebf2* is most strongly associated, and aging-hallmark levels. Together, these results support an inverse association between saddlebrown module activity and aging-hallmark levels and are consistent with the possibility that *Zmiz1* negatively influences this *Srebf2*-associated module under knockdown conditions. Notably, the combined knockdown group did not show a simple additive pattern in the Ri-saddlebrown module. Instead, Ri-S-Z displayed an intermediate module activity between Ri-*Srebf2* and Ri-*Zmiz1* (Figure 5C), suggesting that simultaneous perturbation of both genes does not merely strengthen the single-gene response. This pattern supports a non-linear interaction between *Srebf2*- and *Zmiz1*-associated networks, in which the two factors partly converge on shared programs but retain gene-specific regulatory effects.

Finally, pathway enrichment analysis of genes within the saddlebrown module revealed significant enrichment in processes relevant to neuronal maintenance, including cholesterol biosynthesis, macroautophagy, and dendrite morphogenesis (Figure 5D). These pathways suggest that the saddlebrown module represents a homeostatic program supporting neuronal integrity. Together, these findings indicate that the saddlebrown module represents an *Srebf2*-linked neuronal maintenance program whose activity is inversely associated with aging-marker levels, thereby providing a network-level basis for the direction-dependent phenotypes observed in earlier assays.

## 4. Discussion

This study identifies *Srebf2* and *Zmiz1* as neuron-intrinsic regulators of a senescence-like program in basal forebrain neurons using an in vitro platform integrating AAV-based perturbation, quantitative immunocytochemistry, and low-input transcriptomic profiling. *Srebf2* showed a bidirectional association with senescence markers, whereas *Zmiz1* acted more directionally, with overexpression promoting and knockdown attenuating senescence-associated changes. In addition, overexpression of either gene increased the other, suggesting a positive interaction that may reinforce aging-related phenotypes. Together, these results extend our prior single-cell findings and implicate *Srebf2* and *Zmiz1* as coupled regulators of age-associated cholinergic vulnerability.

A key phenotypic feature is the U-shaped response to *Srebf2* modulation, in which both overexpression and knockdown increased p16 and p21. These cyclin-dependent kinase inhibitors are master regulators of cell cycle arrest, DNA damage repair, and inflammatory signaling in senescent cells [19]. Their coordinated dysregulation by *Srebf2* suggests a mechanistic link between metabolic stress arising from cholesterol imbalance and cell signaling reprogramming, which together compromise neuronal resilience. This U-shaped response aligns with clinical observations linking dysregulated cholesterol metabolism to accelerated brain aging [20]. This pattern supports the idea that basal forebrain neurons require tight buffering of cholesterol-related homeostasis and that deviations in either direction can engage senescence-associated stress programs.

By contrast, *Zmiz1* behaves more dose-dependently, consistent with a model in which increased *Zmiz1* availability facilitates pro-senescent transcriptional reprogramming, while its reduction partially relieves this state. Apoptotic signaling, assessed by cleaved caspase-3, followed a distinct trend: caspase-3 did not decrease in either knockdown condition but increased in both overexpression conditions. We interpret this asymmetry as a floor effect in healthy, non-aged neurons, in which reducing senescence-associated regulators may not lower caspase activity below baseline, whereas overexpression can promote an upward shift because senescence-like stress and apoptotic pathways are partially coupled.

The present study used p16, p21, and cleaved caspase-3 as focused readouts of senescence-associated and apoptosis-related changes in primary neurons. Because neuronal senescence is a multifaceted state, additional assays such as SA-β-gal activity, γH2AX foci, mitochondrial function, lysosomal activity, and inflammatory or senescence-associated secretory markers would further clarify which specific stress-related processes are engaged by *Srebf2* or *Zmiz1*. Therefore, the present findings should be interpreted as evidence that these genes modulate senescence-associated marker patterns, while broader marker panels will be useful for future mechanistic dissection.

At the molecular level, the effect of *Zmiz1* perturbation on Filipin-labeled cholesterol suggests a previously underappreciated link between *Zmiz1* and neuronal cholesterol homeostasis, although this effect was less pronounced than that of *Srebf2*. Because *Zmiz1* is a transcriptional coactivator rather than a canonical cholesterol-biosynthetic regulator, this effect is likely indirect. One plausible route is through *Srebf2*-related transcriptional programs, as *Zmiz1* overexpression increased *Srebf2* expression in our transcriptomic dataset. Alternatively, *Zmiz1* may influence cholesterol accumulation through broader effects on neuronal stress responses, maintenance pathways, or cholesterol trafficking and turnover. Further analysis of *Srebf2*-responsive genes such as *Hmgcr*, *Hmgcs1*, *Mvd*, *Sqle*, and related cholesterol metabolic regulators will help define the mechanistic connection between *Zmiz1* and cholesterol homeostasis. Low-input transcriptomic profiling further supported disease relevance and specialization. A set of 55 genes changed concordantly across perturbations, increasing under overexpression and decreasing under knockdown, implicating a shared downstream output axis related to cytoskeletal and synaptic integrity. These 55 genes include *Mapt*, *Grin2b*, and *Dnmt1*, whose expression patterns align with established aging signatures in vivo. For example, *Mapt* upregulation and synaptic gene dysregulation are consistent with features of AD-related neuropathology in rodent models [7]. These representative genes are interpreted as components of broader pathway- and module-level signatures associated with *Srebf2* and *Zmiz1* perturbation. Moreover, the enrichment of ATPase/GTPase pathways in overexpression groups resonates with reports of metabolic dysfunction in aged neurons [14]. At the pathway level, *Zmiz1* overexpression preferentially engaged an Alzheimer’s disease-associated gene signature, whereas *Srebf2* was more closely linked to cholinergic synapse-related programs and downstream signaling components.

The reciprocal induction observed under overexpression conditions suggests an asymmetric regulatory coupling between *Srebf2* and *Zmiz1*, but the underlying mechanism is likely not a simple linear feedback loop. One possibility is direct or semi-direct transcriptional cross-activation. *Zmiz1* is a transcriptional coactivator and may enhance *Srebf2* expression through cooperation with other transcription factors, whereas *Srebf2*-driven changes in lipid metabolic state may secondarily influence *Zmiz1* expression through stress-responsive or chromatin-associated programs. A second possibility is that both genes respond to shared upstream regulators activated by neuronal stress, metabolic imbalance, or aging-associated signaling, leading to coordinated upregulation when either factor is experimentally increased. The absence of reciprocal suppression after knockdown further supports a threshold- or state-dependent model, in which elevated *Srebf2* or *Zmiz1* can engage a feed-forward transcriptional program, whereas basal expression of the other gene may be maintained by compensatory or redundant regulatory inputs. Thus, the present data support regulatory coupling between *Srebf2* and *Zmiz1* under overexpression conditions, while future promoter-level, chromatin-binding, and rescue analyses will be needed to distinguish direct transcriptional activation from shared upstream or network-mediated mechanisms.

Within this framework, network analysis provides a system-level explanation for the phenotypic divergence between the two factors. The *Srebf2*-correlated Ri-saddlebrown module was enriched for maintenance pathways such as cholesterol biosynthesis, macroautophagy, and dendrite morphogenesis, consistent with a homeostatic program supporting neuronal integrity. Importantly, module activity, quantified by AUC and enrichment scores, inversely correlated with immunostaining-based senescence marker levels, supporting the interpretation that this module reflects a protective state. Suppression of this module upon *Srebf2* knockdown is consistent with increased senescence markers, while increased module activity upon *Zmiz1* knockdown suggests that *Zmiz1* may antagonize this protective program under gene suppression conditions, although the mechanism remains to be determined. The combined perturbation groups further indicate that the relationship between *Srebf2* and *Zmiz1* is not well explained by a simple additive or synergistic model. Across transcriptomic and module-level analyses, dual perturbation mainly highlighted shared downstream responses and context-dependent convergence, while the Ri-saddlebrown module showed an intermediate response in the combined knockdown condition. Thus, *Srebf2* and *Zmiz1* appear to operate as coupled but non-equivalent regulators, with interactions that depend on perturbation direction and network context rather than producing uniform amplification.

The use of neonatal primary neurons should also be considered when interpreting the aging relevance of this study. Although neonatal neurons do not fully recapitulate adult or aged neuronal states, primary neurons undergo substantial maturation during in vitro culture, including neurite extension and formation of dense neuronal networks. This culture system provides high experimental tractability, efficient viral manipulation, and a controlled environment for isolating neuron-intrinsic responses from systemic metabolic states, glial interactions, and circuit-level compensation. The present model should therefore be viewed as a reductionist platform for testing whether aging-upregulated candidate genes can induce senescence-associated marker patterns, rather than as a complete model of brain aging.

The transcriptomic component of this study also requires interpretation in light of dataset composition. Overexpression samples were enriched for cholinergic neurons, whereas knockdown samples represented transduced basal forebrain neurons without cholinergic enrichment. Therefore, the cross-directional overlaps reported here should be interpreted as context-shared responses recurring across related neuronal populations, rather than as strict reciprocal responses within an identical neuronal subtype. Within each dataset, however, comparisons were performed against the corresponding matched control, allowing direction-specific transcriptional and network responses to be evaluated in their own experimental contexts. In addition, although target transcript validation and reporter-based transduction support the effectiveness of the perturbations, rescue experiments and further independent specificity controls would strengthen the assignment of downstream effects to direct *Srebf2*- or *Zmiz1*-dependent mechanisms. These considerations support interpreting the present findings as perturbation-associated regulatory relationships and network-level hypotheses that provide a basis for future mechanistic validation.

This study is limited by the acute and in vitro nature of the perturbations and does not capture chronic, multicellular aspects of brain aging. In addition, the overexpression-specific reciprocal regulation between *Srebf2* and *Zmiz1* may arise from direct cross-activation, shared upstream regulators, or cell-type-dependent responsiveness. Future work using matched neuronal subtypes across perturbation directions, time-resolved perturbations, neuron–glia systems, and in vivo aging models will be essential to establish temporal order and causality.

## 5. Conclusions

In summary, our results support a model in which *Srebf2* and *Zmiz1* jointly shape neuronal aging through coupled metabolic and transcriptional programs. *Srebf2* is linked to a protective module whose activity is associated with reduced senescence markers, whereas elevated *Zmiz1* promotes senescence-associated transcriptional shifts and may repress *Srebf2*-linked resilience at the network level. Their reciprocal upregulation under overexpression suggests a potential amplification route once either factor becomes elevated, providing a regulatory framework for understanding how these aging-upregulated genes may contribute to age-associated neuronal vulnerability.

## Figures and Tables

**Table 1:** Cell flow cytometry grouping scheme.

Type of Modulation	Group	Viral Vectors Used
Overexpression	OE-CT	scrambled shRNA virus
	OE-*Srebf2*	*Srebf2* overexpression
	OE-*Zmiz1*	*Zmiz1* overexpression
	OE-S-Z	*Zmiz1* + *Srebf2* overexpression
RNA interference	Ri-CT	scrambled shRNA virus
	Ri-*Srebf2*	*Srebf2* RNA interference
	Ri-*Zmiz1*	*Zmiz1* RNA interference
	Ri-S-Z	*Zmiz1* + *Srebf2* RNA interference

**Table 2:** Software tools and versions used for analysis.

Software	Version	Main Purpose
Fastqc	v0.12.1	Quality control of raw sequencing data
Trimomatic	v0.39	Filtering and trimming of sequencing reads
Hisat2	v2.2.1	Alignment of sequencing reads to a reference genome
FeatureCounts	v2.0.3	Quantification of gene expression levels
Seurat	5.0.1	Data integration and batch effect correction in RNA-seq analysis
GSVA	1.50.0	Gene set variation analysis
WGCNA	1.72-5	Construction of co-expression networks and module detection
ClusterProfiler	4.10.1	Functional enrichment analysis (GO, KEGG, etc.)
metascape	3.5	Functional annotation and pathway enrichment analysis
cytoscape	3.10.2	Visualization and analysis of gene regulatory networks
ImageJ	1.54s	Quantification of microscopy images

**Table 3:** Antibodies used in the study.

Antibody	Brand	Cat. No.	Concentration
Rabbit anti-p21	Abcam (Cambridge, UK)	AB188224	1:500
Rabbit cleaved caspase-3	Cell Signaling (Danvers, MA, USA)	9661S	1:400
Rabbit anti-NeuN	Abcam (Cambridge, UK)	AB177487	1:500
Mouse anti-p16	Santa Cruz Biotechnology (Dallas, TX, USA)	SC-1661	1:100
Chicken anti-MAP2	Abcam (Cambridge, UK)	AB5392	1:2000
Rabbit anti-HA Alexa Fluor 488	Abcam (Cambridge, UK)	AB305269	1:500
Donkey anti-rabbit Alexa Fluor 594	Life Technologies (Carlsbad, CA, USA)	A21207	1:500
Donkey anti-mouse Alexa Fluor 647	Life Technologies (Carlsbad, CA, USA)	A31571	1:500
Donkey anti-chicken Alexa Fluor 647	Invitrogen (Carlsbad, CA, USA)	A78952	1:500

## Data Availability

All data related to this study are contained within the manuscript. Data can be obtained from the corresponding authors on request.

## Supplementary Materials

The following supporting information can be downloaded at: https://www.mdpi.com/article/10.3390/biology15120938/s1, Figure S1. Functional enrichment analysis of selected *Srebf2*- and *Zmiz1*-associated co-expression modules. (A) Enriched functional terms of overexpression-associated modules. (B) Enriched functional terms of RNAi-associated modules. For each module, the top five enriched terms ranked by FDR-adjusted *p* value are shown. Dot size represents −log_10_ (adjusted *p* value). Modules were selected based on their association with *Srebf2* or *Zmiz1* expression in the co-expression network analysis. Dot color indicates different modules. Figure S2. Top hub genes in selected overexpression- and RNAi-associated co-expression modules. Bar plots show the top 25 hub genes in each selected module ranked by module membership (kME). The x-axis represents module membership, and the y-axis lists gene symbols. Modules were selected based on their association with *Srebf2* or *Zmiz1* expression in the co-expression network analysis.
