# Supplementary material for: Regulation of Neuronal Senescence by Srebf2 and Zmiz1 Reveals Mechanisms of Aging-Related Neurodegeneration
Source: Biology (Basel). 2026 Jun 16;15(12):938. doi: 10.3390/biology15120938 (PMC13295397; doi:10.3390/biology15120938)
Supplement: Supplementary file 1 [file biology-15-00938-s001.zip › biology-4320954-supplementary.pdf]

Supplementary Figures

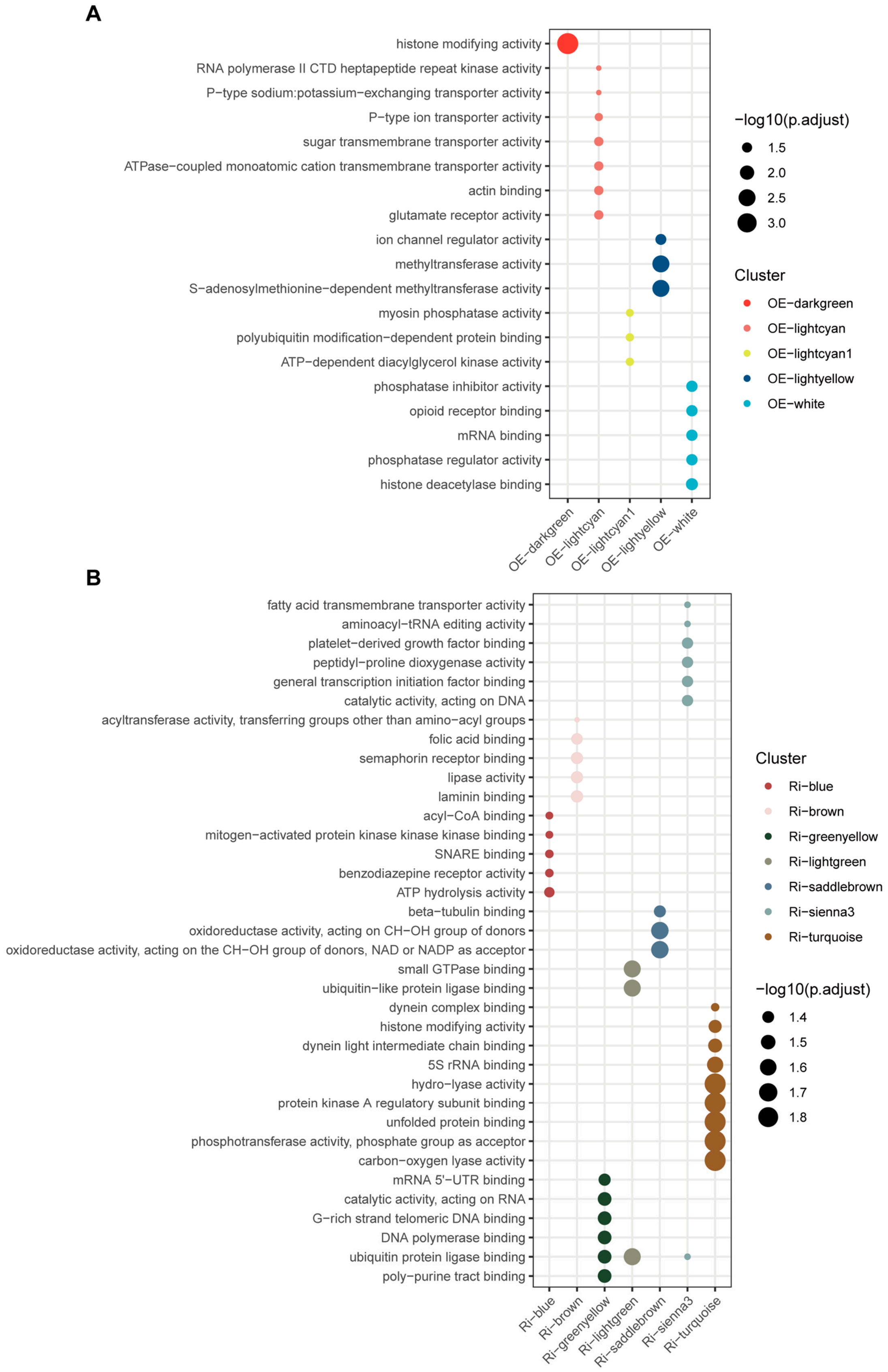

Supplementary Figure S1. Functional enrichment analysis of selected *Srebf2*- and *Zmiz1*-associated co-expression modules. (A) Enriched functional terms of overexpression-associated modules. (B) Enriched

functional terms of RNAi-associated modules. For each module, the top five enriched terms ranked by FDR-adjusted P value are shown. Dot size represents −log10(adjusted P value). Modules were selected based on their association with *Srebf2* or *Zmiz1* expression in the co-expression network analysis. Dot color indicates different modules.

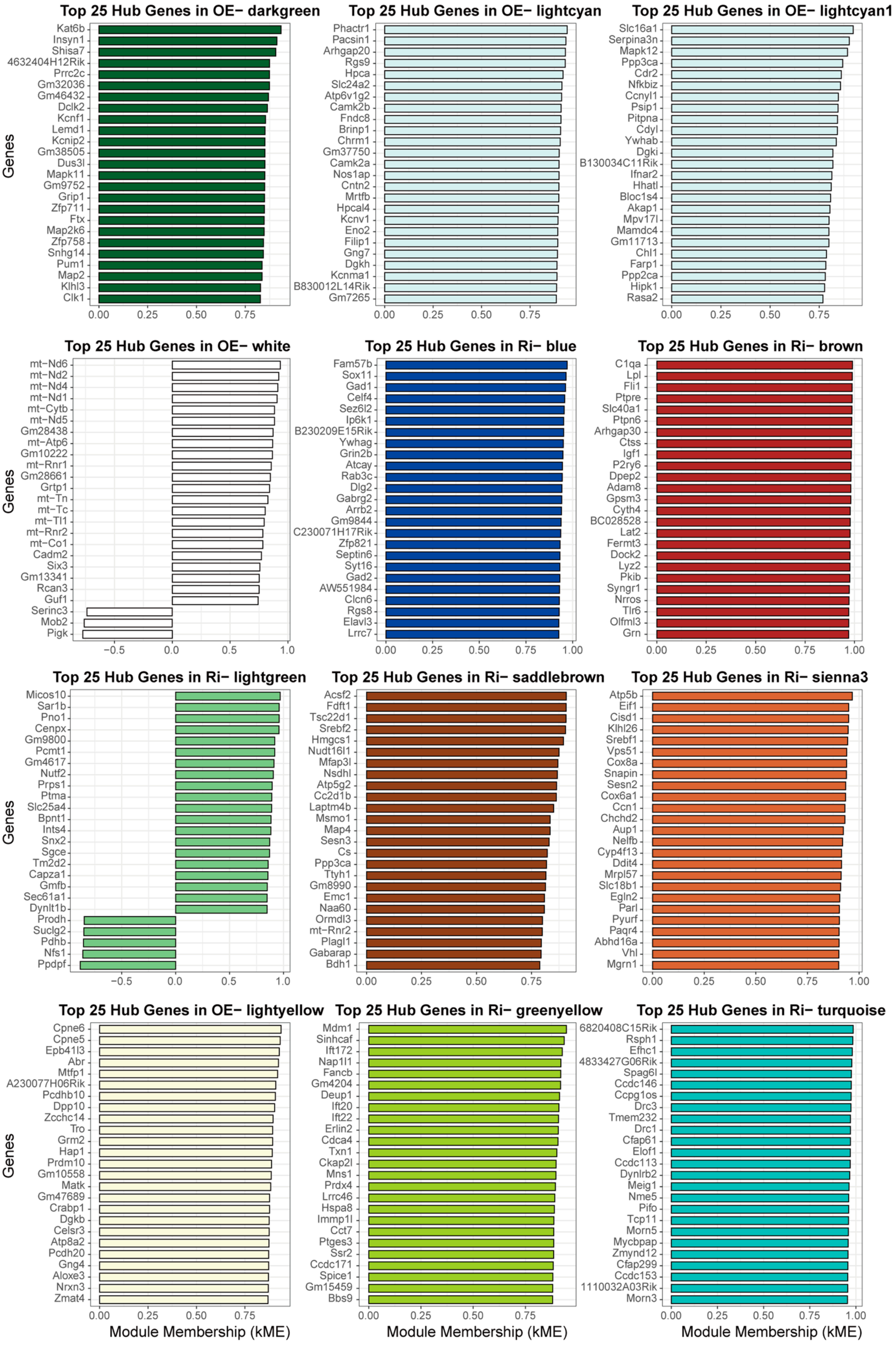

Supplementary Figure S2. Top hub genes in selected overexpression- and RNAi-associated co-expression modules. Bar plots show the top 25 hub genes in each selected module ranked by module membership (kME). The x-axis represents module membership, and the y-axis lists gene symbols. Modules were selected based on their association with *Srebf2* or *Zmiz1* expression in the co-expression network analysis.
